# Atomistic reaction mechanism of CVD grown MoS_2_ through MoO_3_ and H_2_S precursors

**DOI:** 10.1038/s41598-022-20531-x

**Published:** 2022-09-27

**Authors:** Abdullah Arafat, Md. Sherajul Islam, Naim Ferdous, A. S. M. Jannatul Islam, Md. Mosarof Hossain Sarkar, Catherine Stampfl, Jeongwon Park

**Affiliations:** 1 Department of Materials Science and Engineering, Khulna University of Engineering & Technology, Khulna, 9203 Bangladesh; 2 Department of Electrical and Electronic Engineering, Khulna University of Engineering & Technology, Khulna, 9203 Bangladesh; 3 School of Physics, The University of Sydney, Sydney, NSW 2006 Australia; 4 School of Electrical Engineering and Computer Science, University of Ottawa, Ottawa, ON K1N 6N5 Canada; 5 Present Address: Department of Electrical and Biomedical Engineering, University of Nevada, Reno, NV 89557 USA

**Keywords:** Engineering, Materials science, Nanoscience and technology

## Abstract

Chemical vapor deposition (CVD) through sulfidation of MoO_3_ is one of the most important synthesis techniques to obtain large-scale and high-quality two-dimensional (2D) MoS_2_. Recently, H_2_S precursor is being used in the CVD technique to synthesize 2D MoS_2_. Although several studies have been carried out to examine the mechanism of MoS_2_ growth in the presence of sulfur and MoO_3_ precursors, the growth of MoS_2_ in the presence of H_2_S precursor has largely remained unknown. In this study, we present a Reactive molecular dynamics (RMD) simulation to investigate the reaction mechanism of MoS_2_ from MoO_3_ and H_2_S precursors. The intermediate molecules formation, the reason behind those formations, and the surface compositions of MoO_x_S_y_H_z_ during the initial steps of CVD have all been quantified. Surprisingly, a sudden separation of sulfur atoms from the surface was observed in the H_2_S precursor system due to the substantial oxygen evolution after 1660 K. The sulfur detachments and oxygen evolution from the surface were found to have a linear relationship. In addition, the intermediate molecules and surface bonds of MoS_2_ synthesized by MoO_3_ and H_2_S precursors were compared to those of a system using S_2_ and MoO_3_ precursors. The most stable subsidiary formation from the H_2_S precursor was found to be H_2_O, whereas in case of S_2_ precursor it was SO. These results provide a valuable insight in the formation of large-scale and high-quality 2D MoS_2_ by the CVD technique.

## Introduction

Two-dimensional (2D) transition metal dichalcogenides (TMDs) such as MoS_2_, WS_2_, MoSe_2_, MoTe_2_, WSe_2_, and WTe_2_ have recently attracted a lot of attention because of their wide variety of applications in field-effect transistors^1–3^, optoelectronic devices^4–6^, and catalysis^7–10^. In particular, 2D MoS_2_ has so far received the greatest attention due to its unique features including high excitation binding energy, large direct bandgap, strong light-matter interaction, large exciton binding energy and catalytic properties^11^. In addition, monolayer MoS_2_ can be used as a flexible substrate due to its excellent mechanical properties^5,12–14^ and can provide active edge sites for the hydrogen evolution reaction^15^. However, all these promising applications necessitate a large scale and high crystalline quality of MoS_2_. Over the past few years, several methods for the synthesis of MoS_2_ have been developed, including mechanical exfoliation^16–22^ and chemical vapor deposition (CVD)^21,23,24^. Mechanical exfoliation and hydrothermal techniques can produce high-quality MoS_2_, but they are limited by low yield and small MoS_2_ crystal sizes^5,25–28^ . Synthesis of crystalline monolayer MoS_2_ with low cost, high yield, excellent optical, electrical and mechanical properties is highly desirable. The CVD process of MoS_2_ has gained special attention because of its greater possibility to grow excellent quality, mono or few layers MoS_2_ with a limited manufacturing cost^29–31^. Moreover, it is a very suitable method because of the weak Van der Waals interaction in the interlayer and strong covalent interaction in the intralayer of MoS_2_ and thus, the peeling process after the formation of MoS_2_ becomes easier^5,31^.

To obtain large-scale and high-quality 2D MoS_2,_ CVD synthesis via oxidation and sulfurization from α-MoO_3_ through thermal vapor sulfurization (TVS)^32–35^ has received a lot of attention. During this process sulfur precursors are generally used^35,36^ for the sulphidation process, after which MoS_2_ forms from MoO_3_. Several investigations were conducted to understand the reaction mechanism involved in this process^32,37–39^. Apart from sulfur precursors, a highly reactive sulfuric precursor such as H_2_S has been employed for wafer-scale development of MoS_2_ films on several substrates^33–35^. The substitution of solid sulfur with H_2_S gas precursor allows for the formation of vertically grown multilayer structures with chemically reactive edge sites that may have a variety of electrochemical uses^40^*.* Due to the relatively substantial thickness of molybdenum films pre-deposited on insulating substrates and used as the Mo source, previously described growth methods are unable to create monolayer MoS_2_^33,34^_._ Monolayer MoS_2_ also cannot be produced using gold-assisted CVD^35^, which forms a Mo–Au surface alloy during the reaction of Mo(CO_6_) with the Au thin film. To understand the growth kinetics Hong et al.^41^ performed a quantum molecular dynamics simulation examining the effect of H_2_S precursors with and without the presence of hydrogen on CVD grown MoS_2_. This study revealed that with the help of extra hydrogen, some hydroxide sites were formed on the ejected MoO_3_ cluster and then it reduces to Mo(OH)_x_ by forming H_2_O gaseous species. However, the surface chemistry of MoO_3_ for H_2_S precursors during the formation of MoS_2_ and the reasons for the formations of intermediate molecules in the system still remain unknown.

In this study, a new chemical insight was provided into the sulfidation of MoO_3_ in the presence of H_2_S as a vapor phase precursor using reactive molecular dynamics (RMD) simulations. The growth kinetics of the formation of intermediate molecules from H_2_S and MoO_3_ was systematically investigated. The intermediate molecules formed in the system during the CVD process were examined. The effect of oxygen evolution on the H_2_S vapor phase was observed and a comparison of the S_2_ and H_2_S precursor deposition on the surface of the metal substrate was provided. This investigation offers new insights on the synthesis of high-quality CVD grown MoS_2_ on a huge scale using H_2_S as a precursor.

## Results and discussion

The investigation started with solid MoO_3_ and H_2_S molecules as vapor phase precursors present in the 200 Å vacuum region of the simulation box and raising the temperature from room temperature (300 K) to 2800 K. The system with the H_2_S precursors was at first relaxed using the conjugate gradient method and the temperature was controlled using an NVT ensemble with Nose–Hoover thermostat^42,43^ with a damping constant of 25.0 fs. These conditions were similar to Hong et al.^44^ where a reactive molecular dynamics simulation was done on the CVD grown MoS_2_ with S_2_ and MoO_3_ precursor. The system was then examined, and the findings were investigated until 375 picoseconds. The elevated temperature was used to clearly observe the chemical reactions in the system with minimal numbers of timesteps. However, MoO_3_ precursor vaporizes at this high temperature, as its melting point is around 1200K^36,45^. To maintain the solid phase, a one-body spring force was applied independently to all Mo atoms on the MoO_3_ surface. This external spring force prevents Mo from detaching from its original positions, allowing the simulation to extract the majority of the essential reaction events such as the intermediate gas formations and the Mo, S, O, and H bonds. Additionally, it also imitates the effect of MoO_3_ on substates because the conditions are similar to when MoO_3_ is attached to substates such as SiO_2_ or Al_2_O_3,_ where bonds are created between Mo and the substrates. Based on the effect of H_2_S and simulation time, this simulation process can be divided into two parts: (i) reduction of H_2_S molecules at lower temperature and (ii) formation of H_2_O molecules at higher temperature.

**Figure 1:**
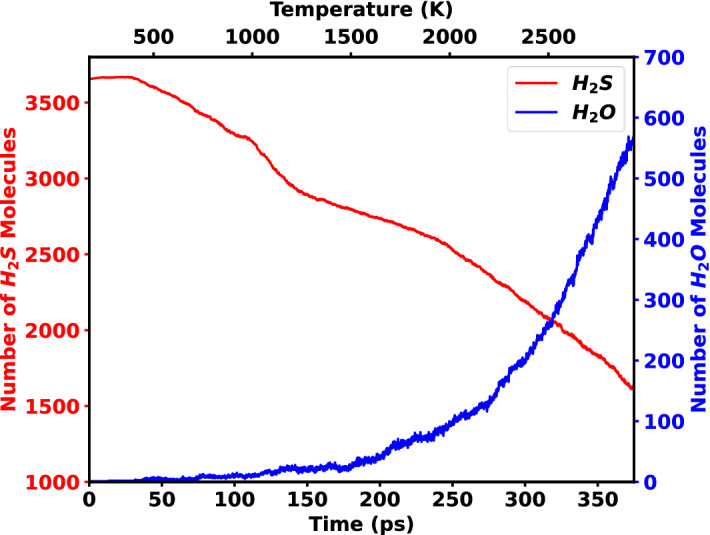
Reduction of H_2_S molecule and formation of H_2_O molecule up to 375 ps in the vacuum. 1968 molecules of H_2_S were reduced and 571 molecules of H_2_O were formed after 375 ps.

The reduction of H_2_S was started at 5 ps and with an increase of the temperature, the reduction increases. This is owing to the features of the H_2_S gas molecules, which are stable at low temperatures but become unstable as the temperature rises. As a result, H_2_S gas particles decompose into many unstable and highly reactive products, such as H_4_S_2_, H_2_S_2_. Some of these unstable compounds recombine and produce H_2_S again. Consequently, the H_2_S curve is less steep in the first half of the simulation as shown in Fig. 1 (red line). Many intermediate molecules were generated in the second portion, including H_2_O, H_2_, HS, SO, and HSO. After 375 ps, 571 molecules of H_2_O, 33 molecules of H_2_, 123 molecules of HS, 137 molecules of SO, and 497 molecules of HSO were formed. This is because the oxygen atoms on the MoO_3_ surface become unstable at high temperatures and evolve into the vacuum. At this point, the previously unstable chemicals generated by H_2_S react with oxygen and stabilize themselves. The most stable molecules created during this process is H_2_O, and it does not transform into any other forms in subsequent phases. Thus, with increasing temperature, other unstable molecules such as HS, HSO, HSO_2_, etc. rapidly transform into H_2_O. This is the reason of the less steep gradient in Fig. 1 (blue line) prior to 200 ps, but rapidly grows thereafter.

To confirm whether the formation of H_2_O is achievable in the aforementioned process, a Nudged Elastic Band–Transition State (NEB-TS) calculation using the density functional theory (DFT) was carried out. As an initial structure, a single MoO_3_ molecule and one H_2_S molecule was taken, and for the final structure, a MoO_2_S with a H_2_O molecule was taken. Initially, the structures were geometrically optimized using the local density approximation (LDA) functional, then the NEB-TS calculation was performed. The NEB-TS was conducted with the image dependent pair potential (IDPP) approach, followed by the Eigenvector Following (EF) method to determine the optimal saddle point.

**Figure 2:**
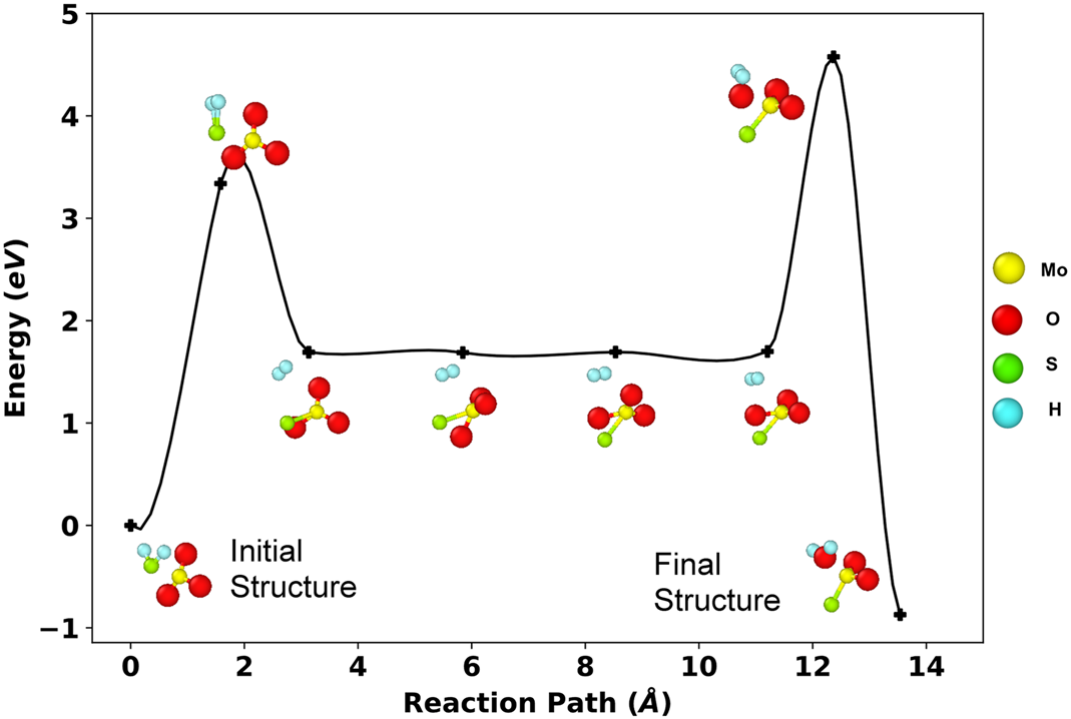
Reaction path and associated energy diagram obtained from Nudged Elastic band-Transition State (NEB-TS) calculations. Here, yellow, red, green, and blue colored spheres represent the molybdenum, oxygen, sulfur and hydrogen atoms, respectively. These colors are used to identify these atoms throughout the whole simulation.

As illustrated in Fig. 2, the NEB-TS calculation reveals that our final structure has lower energy than our initial structure which implies that MoO_3_ + H_2_S → MoO_2_S + H_2_O is chemically possible and MoO_2_S + H_2_O is more stable than MoO_3_ + H_2_S. However, it was also observed that to form H_2_O, the system needs to pass very high energy transition states. This is achievable because the CVD process is caried out at a high temperature. The atoms thus get excited and cross these saddle points. After further investigation into the causes of these transition states, it was determined that two constituents were necessary to overcome the substantial energy barrier, as shown in Fig. 2. These two steps are: (a) formation of MoO_3_S + H_2_ from MoO_3_ + H_2_S and (b) formation of MoO_2_S + H_2_O from MoO_3_S + H_2_. Because H_2_ is also a stable molecule, the system may attempt to produce H_2_ first before attempting to form H_2_O due to the higher second energy barrier. But eventually, with increase in temperature, H_2_O will form. Thus, the DFT NEB-TS calculation implies that the reactions in our MD simulation were valid, and the Reactive force field utilized in our simulation is appropriate to investigate the reaction mechanism of the system.

**Figure 3:**
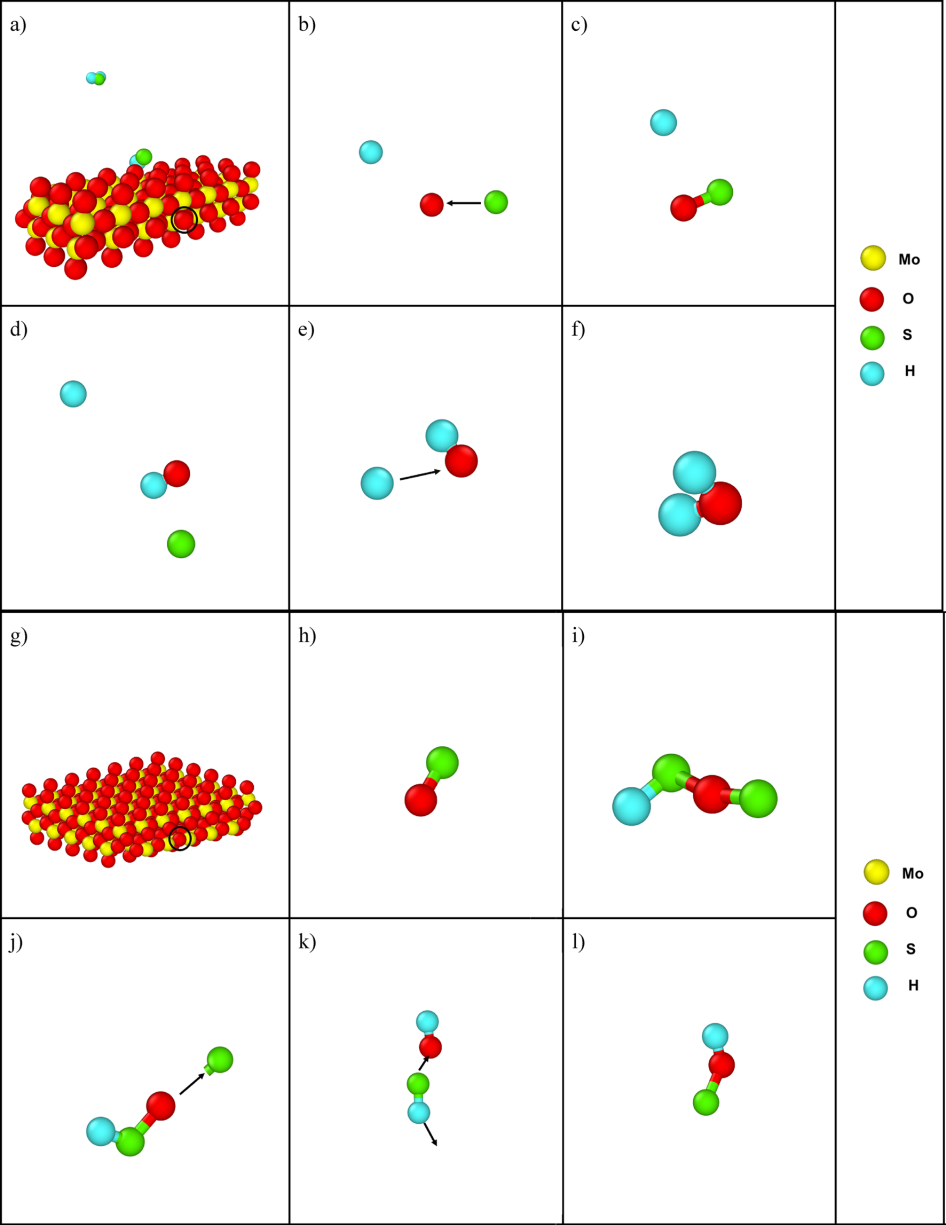
(**a**) MoO_3_ cluster of the first oxygen atom. Snapshot of the first oxygen atom at (**b**) 130 ps, (**c**) 150 ps, and (**d**) 225 ps. (**e**) 260 at (**f**) 280 ps g) MoO_3_ cluster of the second oxygen atom. Snapshot of the first oxygen atom at (**h**) 55 ps, (**i**) 170 ps, and (**j**) 280 ps. (**k**) 300 at (**l**) 375 ps.

Next, rather than focusing on H_2_S molecules, two random O atoms from the MoO_3_ surface were selected and their individual route was analyzed. The trajectory of the first O atom demonstrates that there was little to no movement at first. But after 130 ps, a sulfur atom from an H_2_S molecule comes into contact with the O atom and generates SO, which then evolves into the vacuum and comes closer to a H atom at 150 ps. This H atom interacts with SO atoms to form OH + S at 225 ps. This OH then gets close to another H atom and finally stabilizes as H_2_O at 280 ps. This is because H_2_O is more unstable than OH and reacts faster to form stable compounds, which is shown in Fig. 3a–f. When the second O atom was observed, it was discovered that it followed a different path from the first one. This O atom moves faster away from the surface at first, then combines with a single sulfur atom to generate SO. The SO then reacts with a HS to generate HS-O-S at 170 ps. Later, at 280 ps, the S from HS-O-S breaks, forming HS-O, and then at 290 ps, a new hydrogen atom comes into contact with the HS-O and forms HS + OH, breaking the bond of S and O. Then, at 300 ps, a new sulfur atom from a HS comes into contact with OH and forms SOH, breaking the bond of SH. And finally, it remains as SOH, as seen in Fig. 3g–l. Even though the end product of both of these O atoms was different, an interesting phenomenon was seen when any H atom comes into contact with O. It is most likely that the sulfur gets detached from O and forms OH. But again, when OH is already formed and a new sulfur atom comes into contact, then SOH is formed, and stability prevails. The reason behind this is that the bond of S–O is loosely attached, and the bond is so weak that at elevated temperatures when O moves towards new H, the bond between O and sulfur breaks. On the other hand, as the OH bond is more stable and O has an open end to bond with another atom, so when new sulfur comes into contact with this OH it forms SOH.

**Figure 4:**
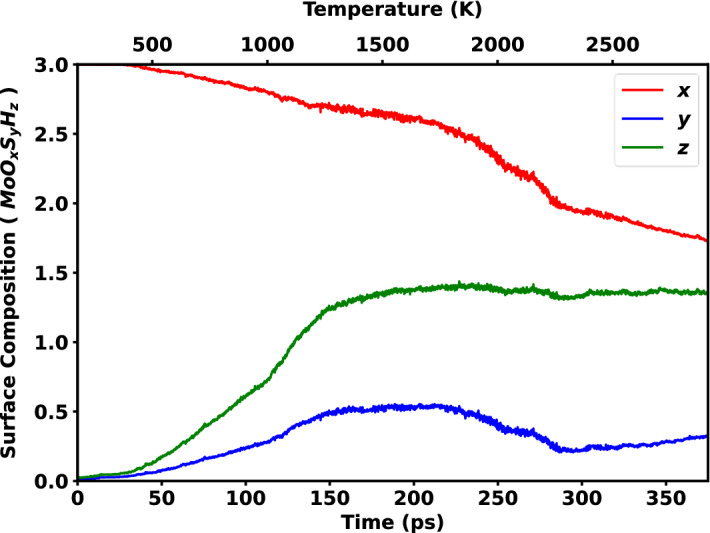
Rate of reduction process. The red, blue and green curves represent the changes in composition of oxygen, sulfur and hydrogen atoms on the surface on the MoO_x_S_y_H_z_ surface. The reduction was observed until 375 ps raising the temperature up to 2800 K.

By focusing on the surface of MoO_3_, some insights into the creation of MoS_2_ were obtained. The surface composition was discovered to be MoO_1.72_S_0.33_H_1.37_ after reaching a temperature of 2800 K at 375 ps. This strongly suggests that the sulfidation and reduction processes took place during the temperature rise. The overall surface chemistry time can be divided into four sections: (a) 0–150 ps, (b) 150–210 ps, (c) 210–280 ps, and (d) 280–375 ps. The amount of sulfur and H on the surface increases up to 150 ps, whereas the amount of O decreases due to O evolution. The oxygen starts to evolve at 75 ps when the temperature was 795 K, which agrees with other studies regarding the melting point of MoO_3_. After that, up to 210 ps, the sulfur content on the surface remains almost constant. This indicates that no new sulfur atoms are becoming bonded to the surface. This is because there are no extra vacant sites left for sulfur to bond with Mo, as very few oxygen atoms are evolving at this temperature. Similarly, the reduction in oxygen atoms from the surface at this time is lower due to the strength of Mo–O bonds at lower temperature. Also, at this time the hydrogen content of the surface becomes constant and does not change anymore throughout the whole process. This phenomenon can clearly be seen from Fig. 4.

**Figure 5:**
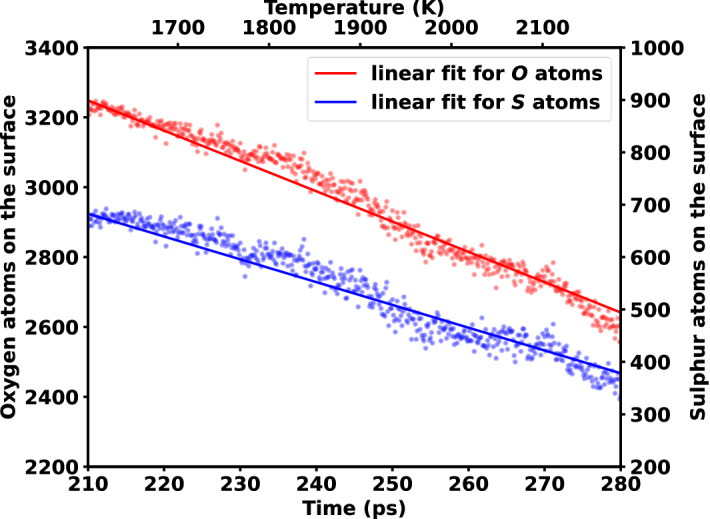
Linear regression fit from 210 to 280 ps over the number of oxygen and sulfur atoms on the MoO_x_S_y_H_z_ surface. Here, blue and red lines represent the linear fit and dotted spheres represent the number of sulfur and oxygen atoms, respectively.

**Figure 6:**
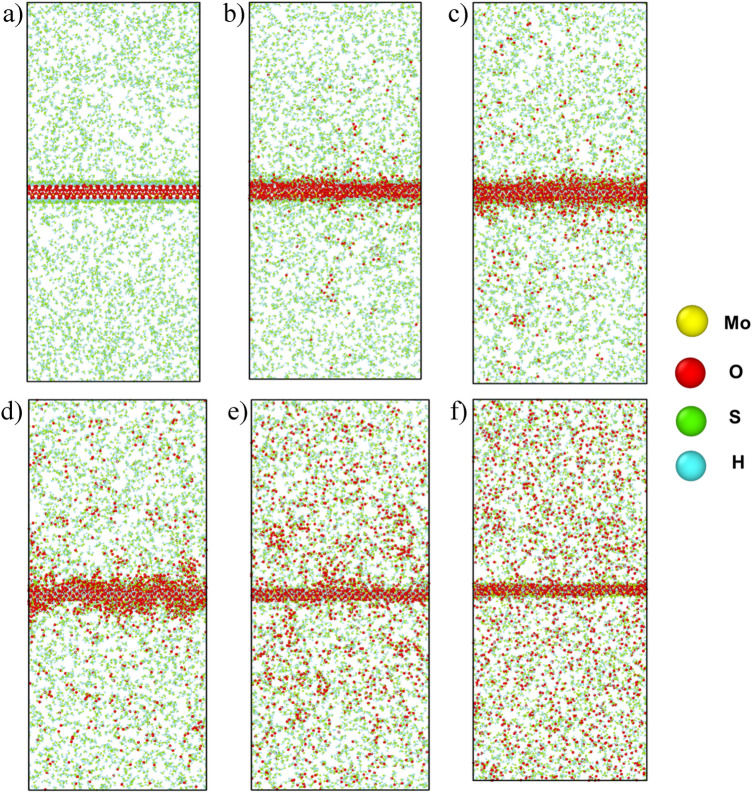
Snapshots of the simulation system at (**a**) 25 ps, (**b**) 95 ps, (**c**) 165 ps, (**d**) 235 ps, (**e**) 305 ps, and (**f**) 375 ps where the surface compositions were MoO_2.99_S_0.03_H_0.05,_ MoO_2.84_S_0.21_H_0.55,_ MoO_2.65_S_0.52_H_1.30_, MoO_2.47_S_0.48_H_1.40_, MoO_1.94_S_0.24_H_1.36_, and MoO_1.72_S_0.33_H_1.37_, respectively.

However, an interesting phenomenon was noticed after 210 ps to 280 ps. The sulfur atoms of the surface start to reduce at this time, which was not expected to happen. At the same time, a steeper descending curve was seen in the oxygen composition from Fig. 4, but no change in surface hydrogen was observed. Initially it was suspected that this phenomenon is occurring due to the evolution of oxygen. But, to justify this, a small portion of Fig. 4 was extracted from 210 to 280 ps and was analysed in Fig. 5. Only the surface composition of oxygen and sulfur was taken, as hydrogen remained constant during this time. Then both the plots were linearly fitted and it was found that the slope of the fitting line of oxygen evolution, m_o_ = −8.677 and the slope of the fitting line of sulfur detachment, m_s_ = −4.34 possess a linear relation m_o_ ~ 2 ms. It was seen that the detaching of sulfur is directly proportional to the evolution of oxygen. But this does not explain how they are affected. To understand this phenomenon, some snapshots of the simulation at different timesteps were taken as shown in Fig. 6. After investigating the images, it was seen that the oxygen atoms start to leave the MoO_3_ surface as early as 75 ps, thus few vacant oxygen sites were produced, and the sulfur atoms subsequently bonds with terminal oxygen atoms. Nevertheless, at 210 ps a sudden increase of oxygen evolution occurs, this is due to the high kinetic energy generated on the surface for elevated temperatures and affording oxygen–oxygen collisions. As a result, a large drop in the sulfur content on the surface was observed, and the previously oxygen bonded sulfur atoms went back to the vacuum with this oxygen evolution. This, in turn, is the cause of the abrupt sulfur composition drop on the surface. After 280 ps, the reaction tends to act normally, with an increase in sulfur content and an increase in oxygen evolution visible on the surface.

**Figure 7:**
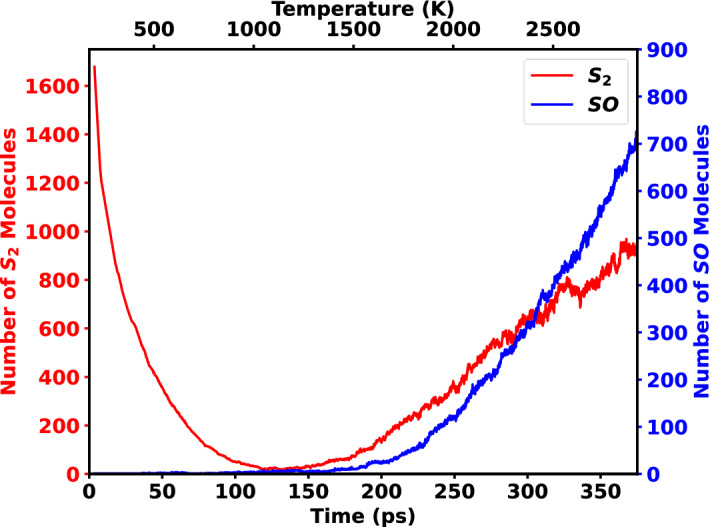
Change in the number of S_2_ molecules and the formation of SO molecules up to 375 ps in the vacuum region of the simulation box. 951 molecules of S_2_ remained and 716 molecules of SO were formed after 375 ps.

In the second part of this study, instead of H_2_S and MoO_3_, S_2_ were considered as initial reactants. With 1800 S_2_ precursor molecules in the vacuum region the temperature was raised from 300 to 2800 K in 375 ps. Upon investigating the behavior of S_2_ precursors with temperature in the vacuum region, it was observed that mostly SO molecules were found instead of S_2_. Moreover, one unpredictable result was also observed that is the sudden fall of S_2_ in the initial steps, as shown in Fig. 7. The amount of S_2_ molecules in the vacuum region decreases dramatically up to 150 ps, while no new SO molecules develops. However, after 150 ps, the quantity of both SO and S_2_ molecules in the vacuum increases.

**Figure 8:**
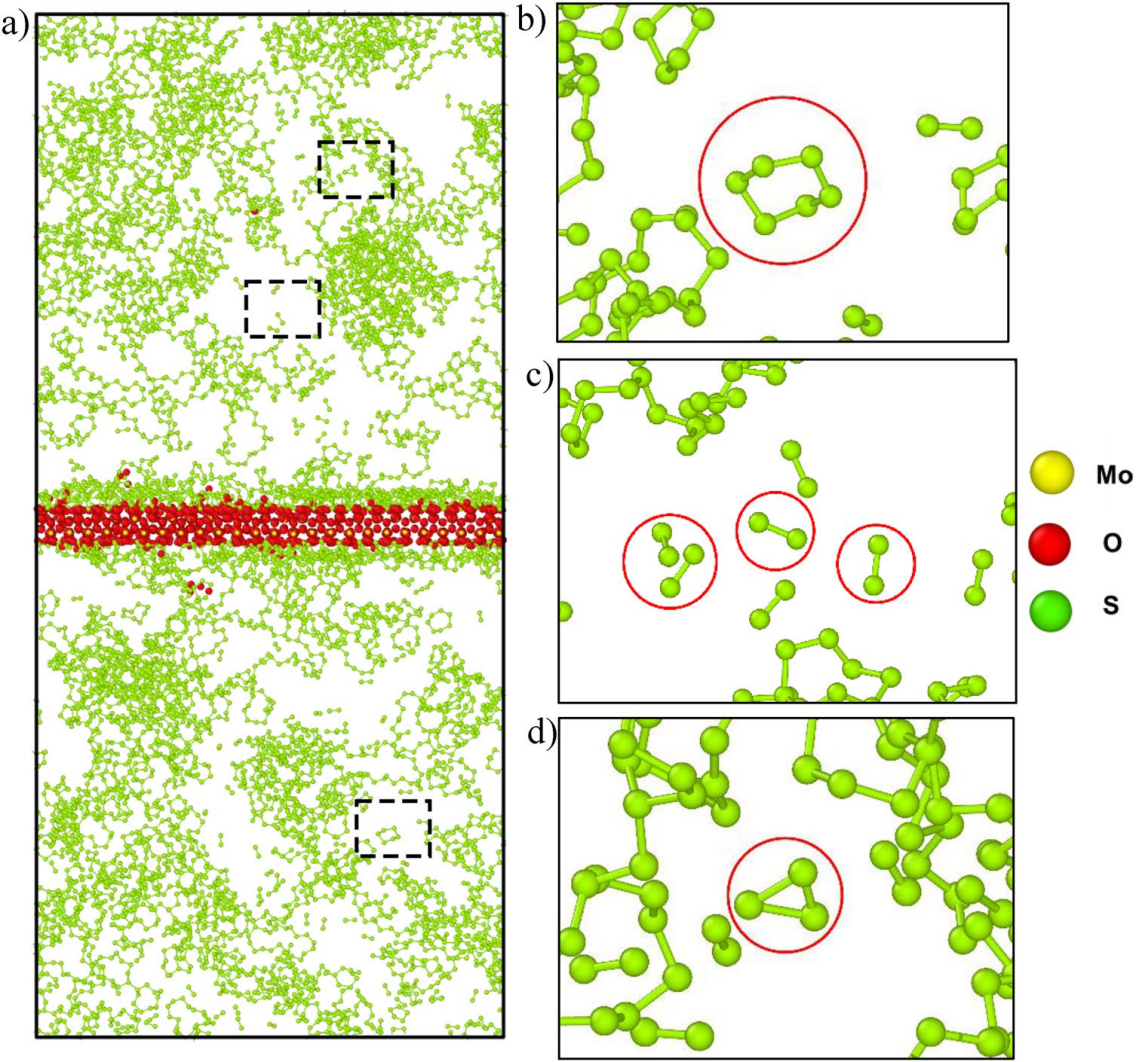
Structure evolution due to the reduction of S_2._ (**a**) Snapshot of the simulation at 50 ps and the formation of (**b**) S6, (**c**) S2, and (**d**) S3 chains.

Upon investigating the structure, it was seen that the initial reduction of S_2_ created several sulfur chains in the vacuum rather than converting itself to any other complexes, as shown in Fig. 8. Eventually, after 125 ps these sulfur chains also revert to S_2_ molecules and remain in the vacuum. This formation of the sulfur chain is due to the effect of temperature. At lower temperatures, S_2_ is not stable but sulfur chains are stable. Whereas at higher temperatures sulfur chains are not stable and breakdown to S_2_ molecule. Interestingly, the formation of this sulfur chain was not found in the H_2_S precursor setup as H_2_S was initially more stable. As a result, no sulfur atoms dissociated from H_2_S formed sulfur chains. On the other hand, from the Fig. 7 it can also be seen that the formation of SO starts after passing 150 ps, which is at around 900 K. At this temperature, oxygen starts to evolve from the surface of the MoO_3_, where it combines with sulfur atoms to create SO. This causes the gradual increase in the SO formation in the system after 900 K.

**Figure 9:**
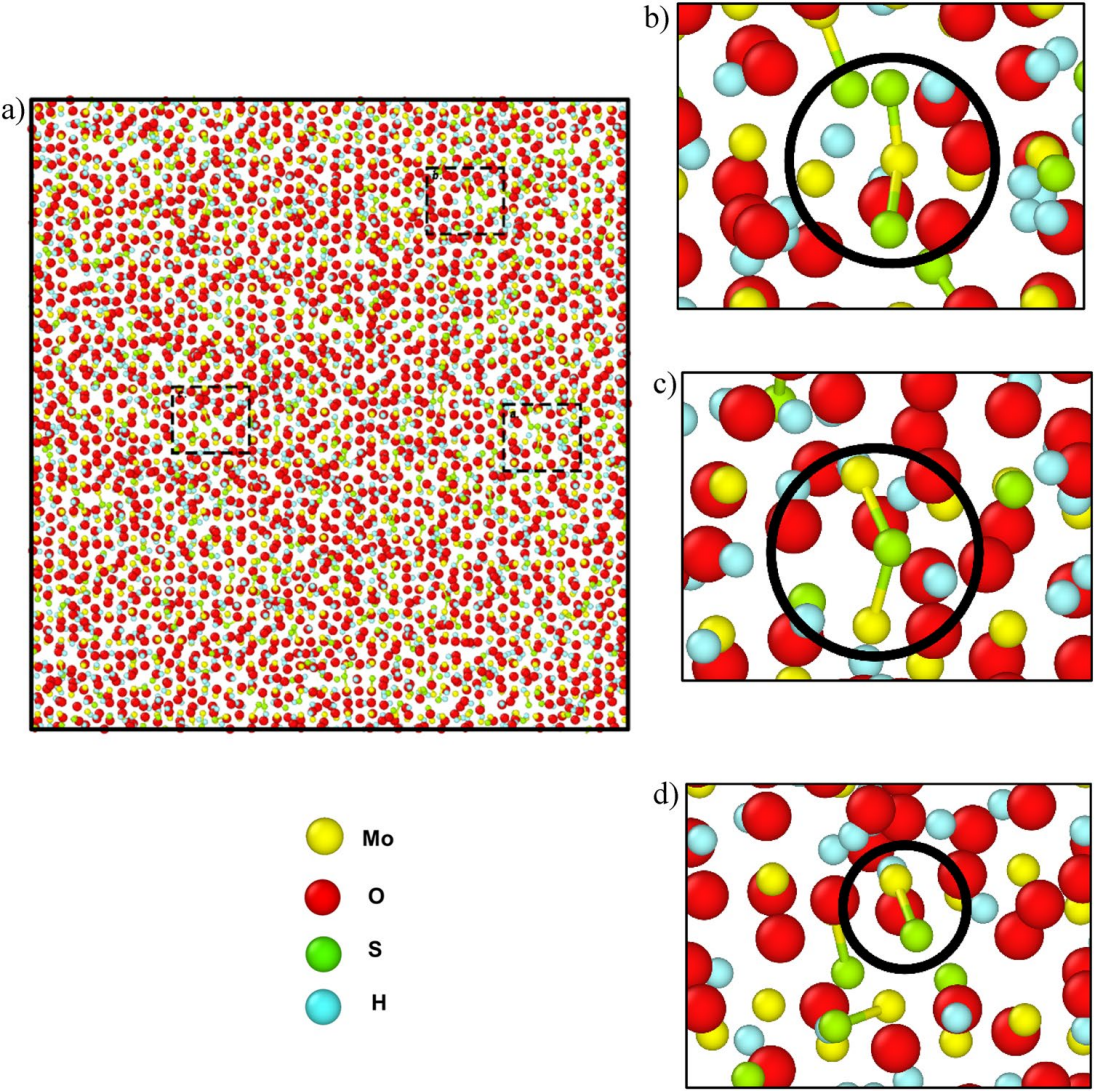
The surface composition due to the H_2_S precursor at 375 ps. (**a**) The formation of the MoO_1.72_S_0.33_H_1.37_ surface showing the enlarged regions highlighting the (**b**) MoS_2,_ (**c**) (Mo)_2_S, and (**d**) MoS bonds.

**Figure 10:**
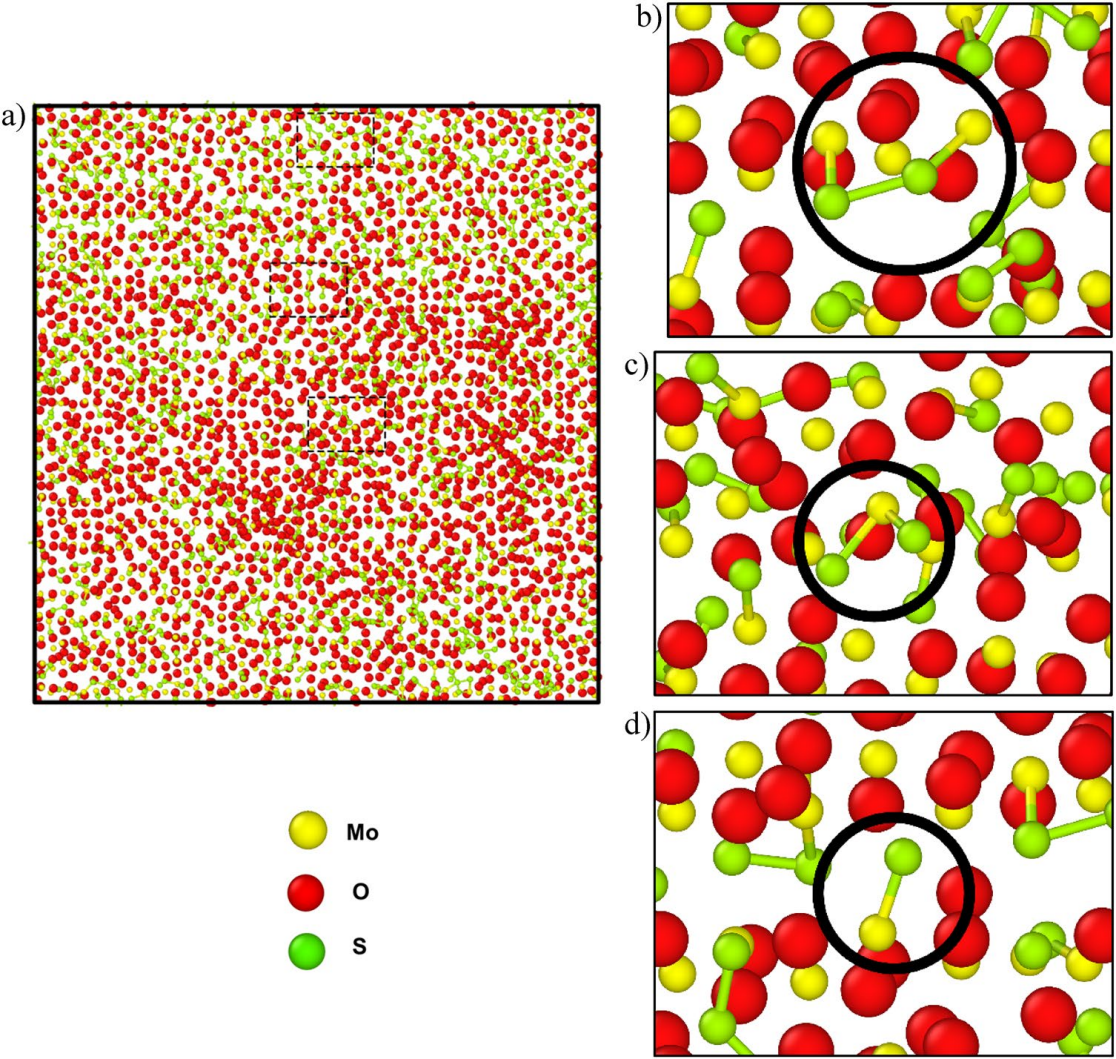
The surface composition due to the S_2_ precursor at 375 ps. (**a**) The formation of the MoO_2.01_S_1.27_ surface showing the enlarged regions highlighting the (**b**) Mo-S_2_-Mo, (**c**) MoS_2_, and (**d**) MoS bonds.

The surface structures were investigated after both systems were run for 375 ps. The surface composition of the H_2_S precursor was MoO_1.72_S_0.33_H_1.37_, and the surface composition of the S_2_ precursor was MoO_2.01_S_1.2_. In addition, MoS_2,_ (Mo)_2_S, MoS, etc. bonds were formed for H_2_S precursor systems and Mo-S_2_-Mo, MoS_2_, MoS, etc. bonds were formed in S_2_ precursor systems which are depicted in Figs. 9 and 10, respectively. This suggests that the bond formation in both the systems are quite similar in nature. And among them most prominent bonds were MoS_2_ and Mo-S bonds. On the other hand, the surface of the S_2_ precursor system appeared to be much closer to MoS_2_ than the surface of the H_2_S precursor system. But the H_2_S vapor phase precursor system provides a closer look at the actual CVD synthesis process. Because hydrogen is already present in the environment, so even if sulfur powder precursors are used then it reacts with the environmental hydrogen and eventually forms H_2_S.

**Figure 11:**
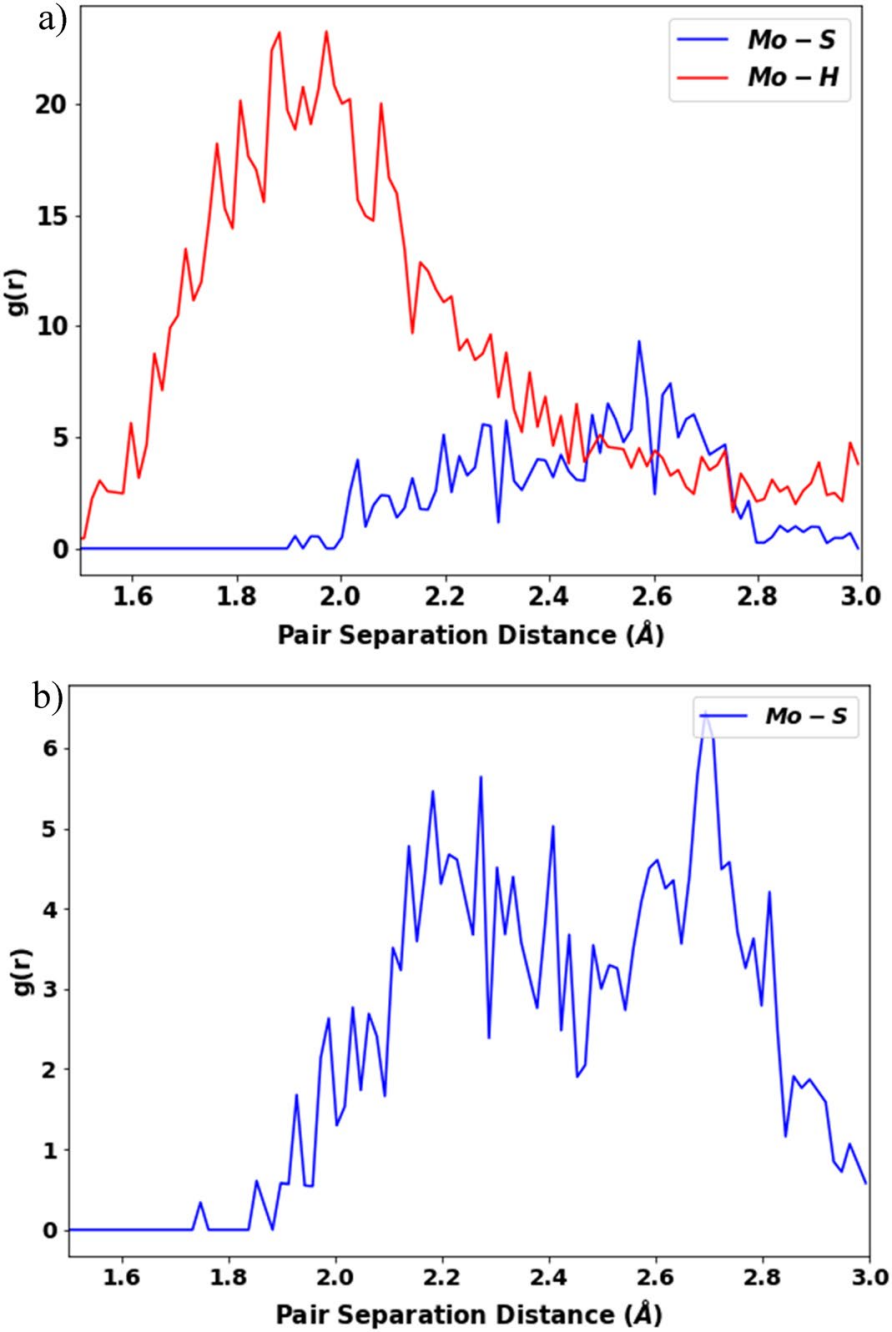
Radial distribution function of (**a**) Mo-S and Mo-H bonds of the H_2_S precursor system and (**b**) Mo-S bonds of the S_2_ precursor system at 375 ps.

After analyzing the radial distribution functions for both systems, it is observed that the density of the Mo-S is maximum for a pair separation distance of 2.5–2.7 Å in the H_2_S system, with values ranging from 2.0 to 3.0 Å. And the density of Mo-H is a maximum around 1.8–2.0 Å, and varies from 1.5 to 3.0 Å (Fig. 11a). In the S_2_ system, on the other hand, the Mo-S reaches its maximum at a pair separation distance of 2.6–2.8 Å, with values ranging from 1.9 to 3.0 Å (Fig. 11b). It implies that the systems are quite similar to the synthesis of MoS_2_. However, experimental studies show that Mo-S has a bond separation distance of 2.31 Å, which is lower than our measured values; this difference is due to the elevated temperature. As our systems were exposed to a much greater temperature than room temperature, therefore due to bond vibration and atom collision at this higher energy state changing the pair separation range can be considered normal.

Additionally, earlier studies by Hong et al.^41,44^ showed how the addition of H_2_ affects the CVD growth of MoS_2_ in the presence of H_2_S precursors and suggested that H_2_ carrier gas acts as an effective reducing agent for the MoO_3_ slab at a constant temperature of 2500 K. An insight into the formation of MoS_2_ surfaces in the presence of the S_2_ precursor was also given by their study^44^. Whereas, in this study, the effect of the H_2_S precursor in changing temperature was discussed in detail, and the intermediate molecules formed in the process were analyzed. The changes in the surface composition and the formation of sulfur clusters in the vapor phase due to the rising temperature were discussed. Although a recent study by Rajak et al*.*^46^ has shown the reaction pathways in the presence of H_2_S, S_2_, and H_2_ as a function of temperature leading to the growth of MoS_2_, they are yet to provide the through-reaction mechanism of the formation of intermediate molecules in the vapor phase, which is the main focus of the present research**.** We investigate the bond formation mechanism of Mo, S, O, and H in the vapor phase, which gives an insight on how the vapor phase changes in the presence of different precursors. Moreover, we also showed how the evolution of oxygen atoms is linearly dependent on sulfur detachment from the surface at rising temperatures. Finally, this research gives an in-depth insight into the chemical reactions that occur during the process of making MoS_2_.

## Conclusion

In conclusion, the formation mechanism of MoS_2_ from MoO_3_ and H_2_S precursors is investigated systematically through RMD simulations. The effect of the S_2_ precursor was also investigated and compared to that of the H_2_S system. H_2_O was found to be the most stable subsidiary formation from the H_2_S precursor system, whereas SO was found to be the most stable subsidiary formation from the S_2_ precursor system. Interestingly, a sudden separation of sulfur atoms from the surface was observed in the H_2_S precursor system, and after further examination, it was seen that the cause of this detachment is due to substantial oxygen evolution after 1660 K. The sulfur separation and oxygen evolution in the surface were found to have a linear relationship. It was also discovered that in the S_2_ system the S_2_ is unstable at lower temperatures and generates sulfur chains, but as the temperature rises, it stabilizes back to S_2_. These findings are important to understand the growth mechanisms of CVD synthesized MoS_2_ using MoO_3_ and H_2_S precursors and pave the way of obtaining the large-scale and high-quality MoS_2_.

## Methods

The system consists of a single layer α-MoO_3_ (001) surface of space group Pnma, placed inside a 200 Å vacuum space (This 200 Å vacuum space was used to imitate the real CVD process) filled with H_2_S precursors and S_2_ precursors, respectively. The simulation box used in the system was orthogonal with a dimension of 94.0256 Å × 99.2326 Å × 200.0 Å. The lateral dimension of monolayer MoO_3_ (5000 atoms) was the same as the simulation box with a thickness of ~ 6.0 Å. The MoO_3_ layer was placed exactly in the middle of the box keeping 100 Å vacuum on both sides along the y axis. Here, αMoO_3_ was considered due to the lower initial system temperature. Periodic boundary conditions (PBCs) were imposed in all directions. All the unit cells of the structures were collected from the materials project^47^ and changed accordingly using VESTA^48^. To understand the surface interaction the optimized ReaxFF by Hong et al.^44^ was utilized. These ReaxFF reactive force field parameters were successfully applied to previous studies^44^ for capturing the CVD process. Out of other forcefield parameters, ReaxFF was used because it can explain the dynamics of systems with complex interfaces^49^ and surface-gas interactions^50–52^ on atomic length scales. ReaxFF was also used in the CVD growth of graphene, *h-*BN and other 2D materials.

Reactive force field divides the system energy into various partial energy contributions, as demonstrated by Eq. (1).

1 $${E}_{system}= {E}_{bond}+{E}_{over}+{E}_{under}+{E}_{val}+{E}_{pen}+{E}_{tor}+{E}_{conj}+{E}_{VdWaals}+{E}_{Coulombs}$$

where *E*_bond_, *E*_over_, *E*_under_, *E*_val_, *E*_pen_, *E*_tor_, *E*_conj_, *E*_VdWaals_, *E*_Columbs_, are partial energies for bond, over coordination, under coordination, valence angle, penalty, torsion, conjugation, van der Waals, Coulomb, respectively. Due to its large amount of partial energy terms, the bond breaking and bond formation of complex systems can be calculated by ReaxFF. It is mainly based on bond order contribution which in term determines partial energies. The bond order term used in ReaxFF is demonstrated by Eq. (2).

2 $${BO{^{\prime}}}_{ij}={e}^{\left[{p}_{bo,1}{\left(\frac{{r}_{ij}}{{r}_{o}}\right)}^{{p}_{bo,2}}\right]}+{e}^{\left[{p}_{bo,3}{\left(\frac{{r}_{ij}^{\pi }}{{r}_{o}}\right)}^{{p}_{bo,4}}\right]}+{e}^{\left[{p}_{bo,5}{\left(\frac{{r}_{ij}^{\pi \pi }}{{r}_{o}}\right)}^{{p}_{bo,6}}\right]}$$

where, *r*_ij_ is the interatomic distance *P*_bo,1_ and *P*_bo,2_ are sigma bonds, *P*_bo,3_ and *P*_bo,4_ are first pi bonds, *P*_bo,5_ and *P*_bo,6_ are second pi bonds. To calculate the species formed in this simulation reax/c/species command was used, which computes the average bond order on large number of timesteps for species analysis. The reactive molecular dynamics simulation was conducted using LAMMPS^53^ with a 0.25 fs integration timesteps.

## Simulation details

The energy of the system was first minimized using the conjugate gradient (CG)^54^ technique, and then the charge equilibration was used to reduce the electrostatic energy using the ReaxFF force field. The equation of motion in the RMD simulation was numerically solved using the velocity Verlet^55^ scheme. After both the systems were relaxed by CG technique, 3600 H_2_S molecules were inserted in the vacuum space of the system. The MoO_3_ layer was constrained using a spring constant of 80 kcal mol^−1^ Å^−1^. After that the system temperature was thermalized from 300 to 2800 K using Nosé-Hoover thermostat for 375 picoseconds with a damping constant of 25.0 fs. Similar conditions were applied in a new system only changing the 3600 H_2_S molecules to 1800 S_2_ molecules. And then data for both the systems were investigated after the simulation was completed. Each RMD simulation was run for 375 ps using a timestep of 0.25 fs.

## Data Availability

The datasets used and/or analyzed during the current study available from the corresponding author on reasonable request.
